# Plasma bile acids are not associated with energy metabolism in humans

**DOI:** 10.1186/1743-7075-7-73

**Published:** 2010-09-03

**Authors:** Gemma Brufau, Matthias J Bahr, Bart Staels, Thierry Claudel, Johann Ockenga, Klaus HW Böker, Elizabeth J Murphy, Kris Prado, Frans Stellaard, Michael P Manns, Folkert Kuipers, Uwe JF Tietge

**Affiliations:** 1 Dept. of Pediatrics, Center for Liver, Digestive and Metabolic Diseases, University Medical Center Groningen, University of Groningen, 9713 GZ Groningen, The Netherlands; 2 Dept. of Gastroenterology, Hepatology, and Endocrinology, Hannover Medical School, 30625 Hannover, Germany; 3 Univ. Lille Nord de France, F-59000, Lille, France; 4 Institut Pasteur de Lille, F-59019, Lille, France; 5 INSERM U1011, F-59019, Lille, France; 6 UDSL, F-59000, Lille, France; 7 Dept. of Gastroenterology, Hepatology and Endocrinology, Klinikum Bremen-Mitte, Bremen, Germany; 8 Kinemed, Inc., Emeryville, CA, USA; 9 Dept. of Medicine, University of California, San Francisco, CA, USA; 10 Dept. of Laboratory Medicine, University Medical Center Groningen, University of Groningen, 9713 GZ Groningen, The Netherlands

## Abstract

Bile acids (BA) have recently been shown to increase energy expenditure in mice, but this concept has not been tested in humans. Therefore, we investigated the relationship between plasma BA levels and energy expenditure in humans. Type 2 diabetic (T2DM) patients (n = 12) and gender, age and BMI-matched healthy controls (n = 12) were studied before and after 8 weeks of treatment with a BA sequestrant. In addition, patients with liver cirrhosis (n = 46) were investigated, since these display elevated plasma BA together with increased energy expenditure. This group was compared to gender-, age- and BMI-matched healthy controls (n = 20). Fasting plasma levels of total BA and individual BA species as well as resting energy expenditure were determined. In response to treatment with the BA sequestrant, plasma deoxycholic acid (DCA) levels decreased in controls (-60%, p < 0.05) and T2DM (-32%, p < 0.05), while chenodeoxycholic acid (CDCA) decreased in controls only (-33%, p < 0.05). Energy expenditure did not differ between T2DM and controls at baseline and, in contrast to plasma BA levels, was unaffected by treatment with the BA sequestrant. Total BA as well as individual BA species did not correlate with energy expenditure at any time throughout the study. Patients with cirrhosis displayed on average an increase in energy expenditure of 18% compared to values predicted by the Harris-Benedict equation, and plasma levels of total BA (up to 12-fold) and individual BA (up to 20-fold) were increased over a wide range. However, neither total nor individual plasma BA levels correlated with energy expenditure. In addition, energy expenditure was identical in patients with a cholestatic versus a non-cholestatic origin of liver disease while plasma total BA levels differed four-fold between the groups. In conclusion, in the various (patho)physiological conditions studied, plasma BA levels were not associated with changes in energy expenditure. Therefore, our data do not support an important role of circulating BA in the control of human energy metabolism.

## Background

Recently, a novel and unexpected role for bile acids (BA) in the regulation of energy metabolism has been reported in mice [1]: addition of the primary BA cholic acid (CA) to a high fat diet prevented body weight gain by increasing energy expenditure and fat oxidation [1]. This effect was explained by plasma BA raising intracellularly active thyroid hormone levels via a G-protein-coupled receptor (Gpbar1/Tgr5)-mediated activation of type 2 iodothyronine deiodinase (D2) in brown adipose tissue [1]. In humans, GPBAR1 and D2 were found to be expressed in white adipose tissue as well as skeletal muscle and BA increased oxygen consumption in cultured human myoblasts [1]. These data suggested that similar (patho)physiological mechanisms in the control of energy metabolism might be operational in humans, but this concept has not yet been tested. Therefore, the aim of our study was to investigate, in patients with different pathologies, whether plasma BA are linked to energy metabolism in humans.

## Methods

Twelve male patients with type 2 diabetes mellitus (T2DM) defined according to the criteria established by the American Diabetes Association [2] and 12 male BMI and age-matched controls were investigated (table 1). The inclusion criteria were: age between 40 and 60 years, and BMI between 25-35 kg/m^2^. Subjects with fasting triglycerides >5.65 mM; HDL-cholesterol <1.55 mM; abnormal TSH or history of thyroid dysfunction; treatment with insulin, thiazolidinediones or BA sequestrants at any time; or treatment with lipid lowering medication within three months of screening were excluded. Diabetes was diet-controlled in 7 subjects and treated with glipizide in 5 subjects. Subjects with fasting glucose >5.5 mM, glucose levels >7.7 mM 2 h after OGTT challenge or fasting insulin >17.0 μU/mL were excluded from the control group. The protocol was approved by the RCRC Institutional Review Board (Austin, TX), and was performed at Diabetes and Glandular Research Associates (San Antonio, TX) and Clinical Pharmacology of Miami (Miami, FL). After the baseline blood sampling, subjects received colesevelam HCl (Daiichi Sankyo, Inc., Parsippany, New Jersey) 3.75 g/d for eight weeks divided into two doses given with lunch and dinner.

**Table 1:** Baseline clinical characteristics.

	colesevelam HCl study		liver cirrhosis study	
	**T2DM**	**controls**	**cirrhosis**	**controls**

	**n = 12**	**n = 12**	**n = 46**	**n = 20**

Age (years)	52.5 ± 1.3	49.0 ± 1.4	48.1 ± 1.3	46.9 ± 2.5
Gender (male/female)	12/0	12/0	26/20	12/8
BMI (kg/m^2^)	31.1 ± 0.8	29.4 ± 1.1	23.0 ± 0.4	23.6 ± 0.9
Cholesterol (mM)	5.0 ± 0.3	4.4 ± 0.2	4.7 ± 0.2	4.9 ± 0.3
Triglycerides (mM)	3.0 ± 0.4	1.4 ± 0.2#	1.0 ± 0.1	1.1 ± 0.1
Glucose (mM)	9.4 ± 0.7	5.0 ± 0.2#	6.2 ± 0.2	4.6 ± 0.1‡
HOMA-IR	6.60 ± 0.98	1.97 ± 1.04#	4.58 ± 0.42	1.80 ± 0.26‡
AST (U/l)	21 ± 2	19 ± 1	37 ± 3	15 ± 1‡
ALT (U/l)	25 ± 2	24 ± 3	32 ± 3	16 ± 1‡
γ-GT (U/l)	n.d.	n.d.	89 ± 10	17 ± 2‡

Data are given as means ± SEM. n.d., not determined. # Significantly different from type 2 diabetic subjects, ‡ significantly different from patients with liver cirrhosis as determined by the Mann-Whitney U-test, at least *P *< 0.05.

In addition, 46 adult patients (26 males/20 females) with histologically-proven liver cirrhosis of varying clinical severity (classified by the Child-Pugh score [3] as Child A: n = 7, Child B: n = 19, Child C: n = 20) due to different etiologies were investigated (viral hepatitis, n = 19; alcoholic, n = 13; primary biliary cirrhosis or primary sclerosing cholangitis, n = 14). All subjects were in a stable clinical condition before entering the study. Subjects with proteinuria, suspected infections, clinically overt diabetes mellitus, thyroid dysfunction, or other endocrine disorder and subjects taking any hormone therapy or beta-blockers were excluded from the study. Patency of portal vein and hepatic artery was documented in patients and controls by Doppler ultrasound. This study protocol was approved by the Ethics Committee of the Medizinische Hochschule Hannover, Germany.

All subjects were studied at rest in the morning after an overnight fast, were thoroughly informed about rationale and possible risks of all procedures, and gave written consent before entering the study.

Resting energy expenditure (REE) was measured using indirect calorimetry as described (colesevalam-HCl study: Sensormedics, Yorba Linda, CA; cirrhosis study: Deltatrac metabolic monitor; Datex Instruments, Helsinki, Finland) [4]. Measured REE values were related to REE values predicted for healthy subjects using the Harris-Benedict formula [5].

BA species were determined by gas chromatography-mass spectrometry as described previously [6,7]. For the cirrhosis study, plasma from 20 healthy control subjects (12 males/8 females) matched to the cirrhosis patients for sex, age and BMI (table 1) was used to establish normal values for BA species in our laboratory.

Statistical analysis was carried out using the non-parametric Mann-Whitney U test (SPSS 16, SPSS Inc, Chicago, IL). *P *values <0.05 were considered statistically significant.

## Results

**Figure 1:**
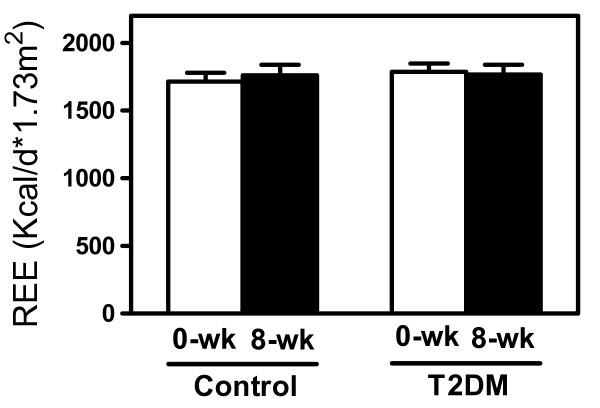
**Resting energy expenditure (REE) in controls and in type 2 diabetic subjects before and after 8-weeks of treatment with colesevelam HCl**. Data are given as means ± SEM.

**Figure 2:**
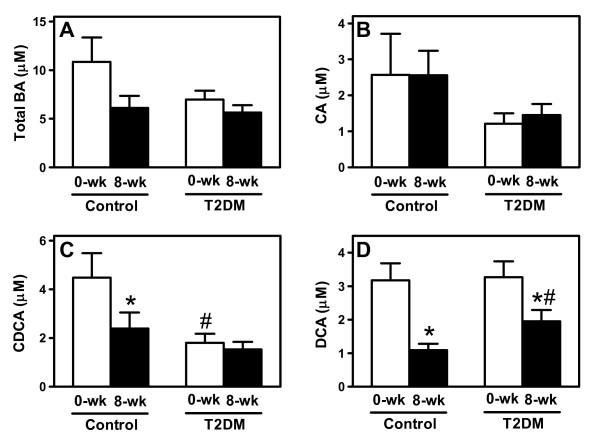
**Plasma bile acid profiles in controls and type 2 diabetic subjects before and after 8 weeks of colesevelam HCl treatment**. (A) Total bile acids, (B) cholic acid (CA), (C) chenodeoxycholic acid (CDCA) and (D) deoxycholic acid (DCA). Data are shown as means ± SEM. *p < 0.05 *vs *baseline and #*p *< 0.05 *vs *controls as determined by the Mann-Whitney U-test.

REE was not different between controls and patients with T2DM before starting treatment (figure 1). Total plasma BA tended to be lower in T2DM due to reduced CA and significantly decreased chenodeoxycholic acid (CDCA) levels (-33%, p < 0.05; figure 2). However, energy expenditure did not correlate with fasting plasma levels of either total or individual BA.

**Figure 3:**
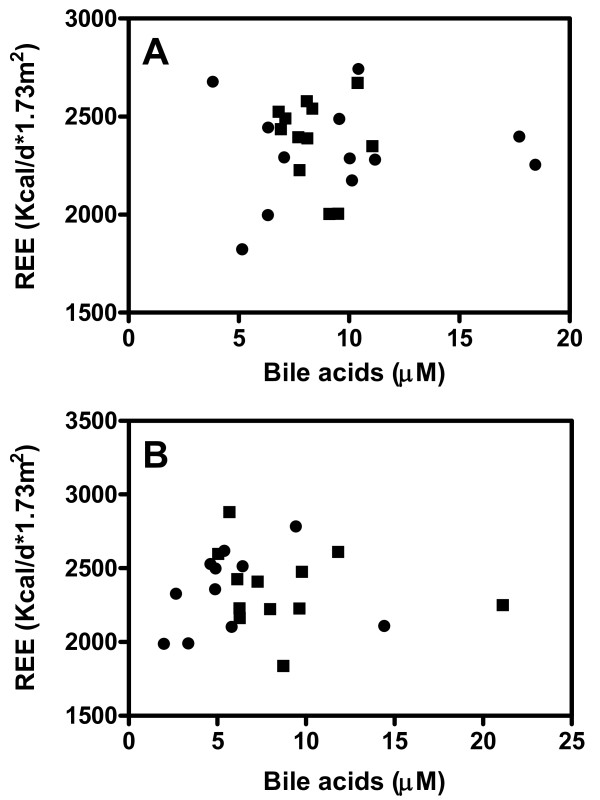
**Correlation between fasting plasma total bile acid levels and resting energy expenditure (REE) in controls (squares) and in diabetic patients (circles) before (A) and after 8-weeks of Colesevelam HCl treatment (B)**. Spearman's rank correlation coefficient was used to assess a possible association between the two different parameters.

Next, we explored the effects of 8-weeks treatment with the BA sequestrant colesevelam HCl on energy metabolism in these subjects. BA sequestrants reduce the flux of BA from the intestine to the liver, thereby reducing plasma BA concentrations, which we hypothesized would translate into changes in energy metabolism. In response to the treatment, DCA levels decreased in both groups (-60% in controls, -32% in T2DM; p < 0.05), while CDCA was only lowered in controls (-33%, p < 0.05, figure 2). In contrast to our hypothesis, colesevelam-HCl did not change REE, and BA levels (total and individual) did not correlate with REE after treatment whether normalized to body surface area (figure 3) or expressed per kg of fat free mass (data not shown).

The second study investigated patients with liver cirrhosis, since these display a varying degree of elevated plasma BA levels and their metabolic state closely resembles the BA-mediated metabolic effects reported in mice: increased REE, increased percentage of energy derived from fat oxidation, and decreased body fat mass (BFM) [5,8-10]. Notably, the underlying pathophysiological basis of these findings is largely unknown, but could conceivably involve BA.

**Figure 4:**
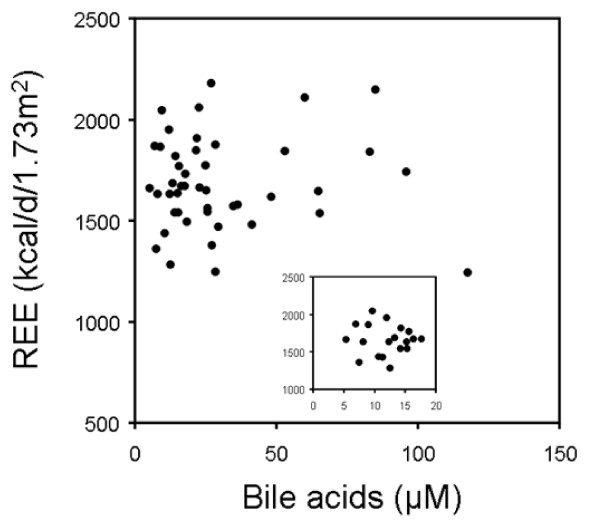
**Correlation between plasma bile acid levels and resting energy expenditure (REE) in patients with liver cirrhosis**. The insert depicts a subgroup analysis including patients with plasma bile acid levels below 18 μM. Spearman's rank correlation coefficient was used to assess a possible association between the two different parameters.

Patients displayed varying degrees of hypermetabolism with an average increase in REE of 18% above the Harris-Benedict prediction (table 2). Total as well as individual plasma BA levels were significantly elevated in cirrhotic patients (table 2). However, neither total plasma BA concentrations (r = 0.049, NS, figure 4) nor individual BA species were correlated with REE. When a subgroup including only subjects with moderately elevated total BA (< 18 μM, n = 19) was studied, REE was still not associated with plasma BA (r = -0.124, NS, insert figure 4). Similarly, subgroup analysis by gender showed no correlation between plasma BA and REE excluding a potential sex-specific effect (data not shown).

**Table 2:** Plasma bile acid levels and energy expenditure in patient groups with liver cirrhosis

	cirrhosis (all) (n = 46)	cholestatic subgroup (n = 14)	non-cholestatic subgroup (n = 14)	normal value
Total BA (μM)	31.2 ± 3.3	40.5 ± 3.4	11.9 ± 1.5	< 10
CA (μM)	10.3 ± 2.2	13.2 ± 0.7	4.2 ± 0.7#	<1.0
CDCA (μM)	13.7 ± 2.9	16.1 ± 4.3	6.7 ± 1.7#	<3.0
DCA (μM)	3.02 ± 1.48	5.81 ± 2.99	0.60 ± 0.16#	<1.0
REE (kcal/d/1.73 m^2^)	1716 ± 33	1676 ± 64	1650 ± 58	
REE (kcal/d/kg FFM)	36.9 ± 0.8	38.5 ± 1.9	37.9 ± 1.3	
REE (% increase)	18 ± 2	19 ± 4	18 ± 3	

Data are given as means ± SEM. BA, bile acids; CA, cholic acid; CDCA, chenodeoxycholic acid; DCA, deoxycholic acid; REE, resting energy expenditure; FFM, fat free mass. The percent increase in REE is the measured value related to the value predicted by use of the Harris-Benedict formula as described in the text. # Significantly different from the cholestatic subgroup as determined by Mann-Whitney U-test, at least *P *< 0.05.

In addition, we compared a subgroup of patients with cholestatic etiology of cirrhosis with greater than 2-fold increased plasma BA (p < 0.01, table 2) to a group with non-cholestatic cirrhotic liver disease exactly matched for sex, age and Child-Pugh stage. However, REE was virtually identical in both patient groups (table 2). Significant differences were also not seen when REE was expressed per kg of fat free mass (table 2).

## Discussion

Our data demonstrate that in different human populations with normal, decreased and variably increased BA concentrations, plasma BA levels are unrelated to energy metabolism. Importantly, lowering of plasma BA levels upon treatment with a BA sequestrant left REE essentially unchanged in controls and in patients with T2DM, a finding that is counterintuitive to BA having a major role in the regulation of human energy metabolism.

Based on data showing an association between circulating plasma BA and energy expenditure Watanabe et. al concluded that in mice, brown adipose tissue (BAT) is the primary target for the metabolic effects of BA [1]. This conclusion is supported by the fact that BAT had the highest relative expression levels of both Gpbar1 and D2 of all mouse tissues investigated [1]. Respective expression levels in human BAT have not been reported, yet [11]. In order to translate the extrahepatic metabolic effects of BA to the human situation, the expression of GPBAR1 and D2 in human skeletal muscle was investigated, but appeared to be very low [1]. Other studies confirmed these results [12] and indicated that the gallbladder is actually the primary site of Gpbar1 expression [13]. This argues against significant BA signaling in human skeletal muscle. In addition, it should be noted that CA and CDCA, major BA species in man, are only poor ligands for Gpbar1 *in vitro *[12]. In our study, plasma concentrations of none of the individual BA species, including one of the strongest Gpbar1 activators DCA [12], correlated with resting energy expenditure. Furthermore, others have shown Gpbar1 knockout mice have no difference in weight gain compared with wild-type mice when fed a CA-containing high fat diet for 9 weeks [13], which was unexpected on the basis of the previous hypothesis [1]. Another group independently generated Gpbar1 knockout mice and observed that feeding a high fat diet without cholic acid for 8 weeks significantly increased body weight and body fat mass, but only in female Gpbar1 knockouts [14]. These results indicate that also in mice the effects of the proposed BA-Gpbar1 signaling axis on energy metabolism are inconsistent.

Additional data arguing against a significant impact of circulating BA on energy expenditure come from studies in obese patients that underwent bariatric surgery. This procedure uniformly results in decreased REE in proportion to weight loss [15,16], while in contrast plasma BA levels increase [17,18].

Since the role of bile acids in the regulation of energy metabolism remains unclear, further studies are warranted. However, our data suggest that there is a chance that GPBAR1/TGR5 agonists, that are currently developed as a novel therapeutic modality against obesity in humans [19], might not be effective.

In summary, we found that in a variety of human settings plasma levels of either total or individual BA were not correlated with energy expenditure. These data suggest that the described metabolic relationship between REE and BA in mice might not be readily translatable into the human situation.

## Competing interests

FK, BS and EJM report receiving research support and consulting fees from Daiichi Sankyo Inc. The other authors declare that they have no competing interests.

## Authors' contributions

GB and MJB were involved in the acquisition and analysis of the data, participated in design and coordination of the study and drafted the manuscript. BS, TC, JO, KHWB and FS contributed to acquisition, analysis and interpretation of data. EJM and KP acquired and analyzed data, participated in the coordination of the study and the critical revision of the manuscript. MPM contributed to interpretation of the data and critical revision of the manuscript. FK and UJFT conceived of the study, participated in its design and coordination, and were involved in critically revising the manuscript. All authors read and approved the final manuscript.
